# Optimizing Aggregate Systems Based on a Binary Paste–Aggregate Model

**DOI:** 10.3390/ma18133047

**Published:** 2025-06-26

**Authors:** Chunming Lian, Xiong Zhang, Lu Han, Weiguo Shen, Lifang Han, Weijun Wen

**Affiliations:** 1 Key Laboratory of Advanced Civil Engineering Materials of Education Ministry, School of Material Science and Technology, Tongji University, 4800 Cao’an Road, Shanghai 201804, China; zhangxiong@tongji.edu.cn; 2 China Construction Eighth Bureau Science and Technology Construction Co., Ltd., 899 Gaoke West Road, Shanghai 201804, China; whosname12@163.com (L.H.); hanlifang0810@126.com (L.H.); 3 State Key Laboratory of Silicate Materials for Architecture, Wuhan University of Technology, Wuhan 430070, China; shenwg@whut.edu.cn

**Keywords:** aggregate mix design, packing density, paste volume minimization, specific surface area, inter-particle spacing

## Abstract

This study presents a systematic method for mix design for optimizing the aggregate proportions in concrete, aiming to minimize paste volume while ensuring adequate workability. Based on a binary paste–aggregate system model, the method refines the calculation of the aggregate packing density by excluding fine particles smaller than 75 μm and incorporating inter-particle interactions across multiple size fractions. A modified approach for calculating the aggregate’s specific surface area is introduced, which accounts for both intra-fraction particle size variation and particle morphology through image-based shape coefficients. Inter-particle spacing is identified as a key control parameter of concrete flowability. Using this criterion, an optimization strategy is developed to determine the ideal aggregate composition that achieves the required spacing with the least amount of paste. Experimental validation confirms that the model reliably predicts paste demand while maintaining desired workability and compressive strength. This physics-based, interpretable approach offers a practical alternative to data-intensive machine learning models and contributes to more sustainable and efficient concrete mix design.

## 1. Introduction

Concrete remains a cornerstone of modern construction due to its versatility, durability, and economic viability [1,2]. However, the environmental burden of cement production—responsible for nearly 8% of global CO_2_ emissions—has underscored the urgent need for more sustainable mix design strategies [3,4]. As the most carbon-intensive and costly component of concrete, cement is a natural target for reduction. Minimizing paste volume, while preserving the required workability and mechanical performance, presents a dual opportunity: lowering both environmental footprint and construction costs.

Traditional concrete mix design relies heavily on empirical rules and iterative laboratory trials, where aggregate proportions are adjusted to meet flow and strength requirements [5,6,7]. These methods often lack generalizability and require increasing the paste content to compensate for suboptimal aggregate packing, leading to excessive binder use and poor resource efficiency [8]. Although packing models such as the compressible packing model (CPM) [9] and wet packing theory [10] have been introduced to rationalize aggregate gradation, they primarily emphasize maximum packing density and often fall short in directly predicting the paste required to achieve target flowability.

More importantly, the existing models rarely offer a unified, quantitative relationship between aggregate morphology, surface area, and the paste volume required to satisfy a prescribed inter-particle spacing—a key parameter governing concrete workability [11]. In addition, fine particles smaller than 75 μm, which strongly influence paste viscosity and rheology, are often ambiguously categorized in binary models, further reducing predictive accuracy [12].

To address these limitations, this study proposes a novel mix design and optimization methodology grounded in the binary paste–aggregate framework. The proposed model simplifies aggregate characterization into two essential, measurable parameters—packing void ratio and specific surface area—and uses these to predict the minimum paste volume needed to maintain a specified inter-particle spacing. This physical model is coupled with a Generalized Reduced Gradient (GRG) optimization algorithm to identify the optimal aggregate proportions that minimize paste content while ensuring workability. The model is experimentally validated using eight concrete mixtures, demonstrating clear reductions in paste demand without compromising flowability or compressive strength.

Compared to conventional approaches and data-driven models, the proposed method presents three key advantages:

(1) Fine particles (<75 μm) are assigned to the paste system rather than the aggregate framework, improving the accuracy of rheological modeling;
(2) Aggregate morphology is quantified using the shape coefficients derived from image analysis, enabling the more precise estimation of the specific surface area;
(3) The inter-particle spacing is explicitly introduced as a design constraint, allowing the paste volume to be directly calculated rather than inferred through trial and error.

Unlike AI-based models that require large datasets and often lack interpretability [13], our physics-based approach offers transparent logic, minimal input requirements, and compatibility with engineering workflows. It also complements data-driven approaches, enabling hybrid predictive schemes that integrate physical understanding with machine learning.

While earlier models such as Kwan’s paste film thickness theory focus on the lubricating function of excess paste through parameters like the WFT, PFT, and MFT [14], they rely heavily on empirical correlations and difficult-to-measure variables. In contrast, our model formulates the problem as a deterministic, constraint-based optimization, grounded in measurable parameters and solvable through numerical algorithms, thus enhancing both usability and sustainability.

The model was validated through experimental trials, including slump flow and compressive strength tests, on mixtures designed using the proposed optimization strategy. The results confirm that the model accurately predicts paste requirements while achieving target performance. Sensitivity analyses demonstrate robustness to variations in aggregate gradation and inter-particle spacing assumptions, further supporting the model’s applicability across diverse concrete mix designs.

In summary, this study introduces a scalable, transparent, and physically grounded framework for concrete mix optimization. By reducing the dependence on excess paste and streamlining aggregate proportioning, the model contributes to the advancement of low-carbon, high-performance concrete. The following sections present the theoretical formulation, optimization methodology, and experimental validation in detail.

## 2. Calculation of Aggregate Packing Density

### 2.1. Compressible Packing Model

“Packing density” is consistently used to refer to the volume fraction of solid particles in an aggregate skeleton. Aggregate gradation is a crucial factor influencing aggregate packing density [15]. While the gradation curve method aims to achieve the densest packing by optimizing aggregate particle size distribution, it does not allow for the direct calculation of the void ratio or provide specific proportions of coarse and fine aggregates [16,17]. During aggregate packing, the interaction between coarse and fine particles is characterized by the “wall effect” and the “loosening effect.” Furthermore, under vibration, the “Brazil nut effect” can occur, leading to segregation [18]. However, in concrete, the viscous resistance of the paste mitigates the Brazil nut effect, resulting in particle packing and spatial distribution that significantly differ from those observed in dry, loose packing.

F. Larrard [9] proposed the Compressible Packing Model (CPM), which accounts for inter-particle interactions and enables the calculation of the void ratio in aggregate systems. When the mixture is dominated by the *i*-th particle size fraction, the packing density is defined as follows:

(1) $$\frac{\beta_{i}}{\gamma_{i}}=1-\sum_{j=1}^{i-1}\left(1-\beta_{i}+b_{ij}\frac{\beta_{i}}{1-\frac{1}{\beta_{i}}}\right)y_{i}-\sum_{j=i+1}^{n}\left(1-a_{ij}\frac{\beta_{i}}{\beta_{j}}\right)y_{j}$$

where $\beta_{i}$ and $y_{i}$ represent the packing density and volume percentage of the i-th particle size fraction, respectively. $a_{ij}$ and $b_{ij}$ are the loosening effect coefficient when coarse particles dominate and the wall effect coefficient when fine particles dominate, respectively. $d_{i}$ and $d_{j}\;$are the particle sizes of the two particles interacting.

(2) $$a_{ij}=\sqrt{1-{\left(1-\frac{d_{j}}{d_{i}}\right)}^{1.02}}$$

(3) $$b_{ij}=1-{\left(1-\frac{d_{i}}{d_{j}}\right)}^{1.50}$$

Due to the complex and variable sources of aggregates used in concrete mixtures, the loosening and wall effect parameters between different particle size fractions are often difficult to determine precisely. When a single particle size fraction is loosely packed, the segregation caused by the “Brazil nut effect” is less pronounced owing to the relatively small size difference among the adjacent fractions. This improves the reliability of experimental packing density measurements, especially for the continuous gradations commonly observed in manufactured aggregates.

To enhance both the accuracy and engineering applicability of the model, the original Compressible Packing Model (CPM), which is typically limited to systems with 2 or 3 discrete particle size fractions, was extended in this study to accommodate 11 particle size intervals, ranging from 26.5 mm to 0.075 mm. This extension allows the model to more accurately represent the continuous grading curves of real-world concrete aggregates. Although the CPM was initially formulated for simplified systems, its core principles—such as the wall effect, loosening effect, and the concept of aggregate compressibility—are not inherently constrained by the number of particle size classes. Prior studies have successfully demonstrated the validity of applying the CPM to systems with six or more fractions, showing good agreement with experimental results [19].

In this implementation, each adjacent pair of particle size fractions is treated as a local two-fraction system, and the global packing density is determined through stepwise application of the CPM equations. The interaction coefficients between particle size classes are computed using Equations (2) and (3), and the detailed values are presented in Table 1. This refined approach preserves the theoretical structure of the original model while enhancing its resolution and predictive capability for complex, multi-fraction grading systems.

The aggregate particle packing process also influences the packing density. Different casting and molding methods result in varying packing density indices:

(4) $$K=\sum_{i=1}^{n}\frac{y_{i}/\beta_{i}}{1/\Phi -1/\gamma_{i}}$$

where Φ represents the actual packing density of the mixed aggregates, calculated using Equation (1). For a given compaction index K, there is a unique packing density, and the packing density of the mixed aggregates can be calculated by solving the equation. Since the packing density of aggregates is related to the workability of concrete, pumping is often employed for the use of ordinary concrete. Based on the empirical findings summarized by F. Larrard, a constant value of 8 is typically adopted for the compaction index K [9].

### 2.2. Aggregate Packing Density Test

**Figure 1:**
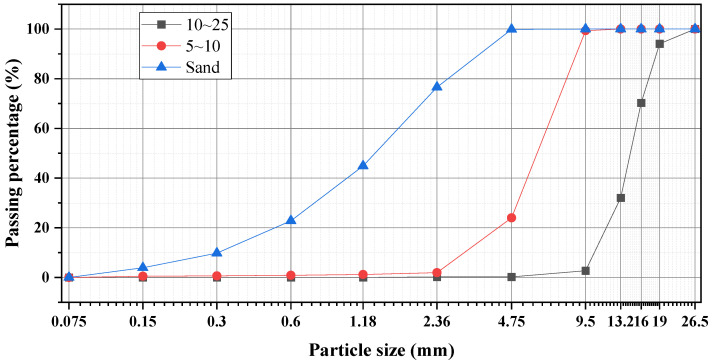
Aggregate gradation curves.

In this study, two fractions of coarse aggregates (5–10 mm and 10–25 mm) and manufactured sand were mixed in different proportions. The packing densities of these aggregate mixtures were tested to validate the accuracy of the model calculations. The particle size distribution of the three types of aggregates is shown in Figure 1.

The packing density of the mixed aggregates was measured using the water displacement method, which minimizes segregation caused by the “Brazil nut effect” through the viscous drag of water. A 10 L cylindrical container was used for testing. First, the empty container was weighed (mass $m_{0}$), then filled with water and weighed again ($m_{1}$). Next, aggregates mixed according to the predetermined proportions were gently poured into the container until the level with the rim, and the mass $m_{2}$ was recorded. The aggregate packing density was then calculated as

(5) $$\Phi =\frac{\left(m_{2}-m_{1}\right)\rho_{w}\rho_{g}}{\left(m_{1}-m_{0}\right)\left(\rho_{g}-\rho_{w}\right)}$$

where $\rho_{w}$ and $\rho_{g}$ are the densities of water and the aggregate particles, respectively.

To ensure measurement reliability, each packing density test was conducted in triplicate for every aggregate mix ratio. The mean value is reported as the result. The coefficient of variation (COV) across replicates was found to be less than 1.8%, indicating excellent repeatability and consistency in the measured values.

To further improve the accuracy of the theoretical modeling and reduce potential errors due to the segregation in mixed grading, each particle size fraction was individually sieved and tested under identical conditions. Table 2 presents the bulk density and stacking compactness for each size fraction. These results were subsequently used as inputs for the CPM-based packing density prediction of mixed gradations.

These measured values not only reflect the morphological diversity and gradation of the aggregates but also form the empirical foundation for calibrating the packing density predicted by the extended CPM under multi-fraction conditions. The results support the model’s validity and provide a reliable basis for paste volume estimation in the subsequent mix design optimization.

### 2.3. Packing Density Calculation Model Validation

The inter-particle interaction parameters adopted from Table 1 were integrated into the improved Compressible Packing Model (CPM). Based on the partial passing percentage of each particle size fraction in the aggregate mix, the theoretical packing density was calculated. To validate the model, 16 combinations of coarse, medium, and fine aggregates were proportioned and tested. The results are presented in Table 3.

**Figure 2:**
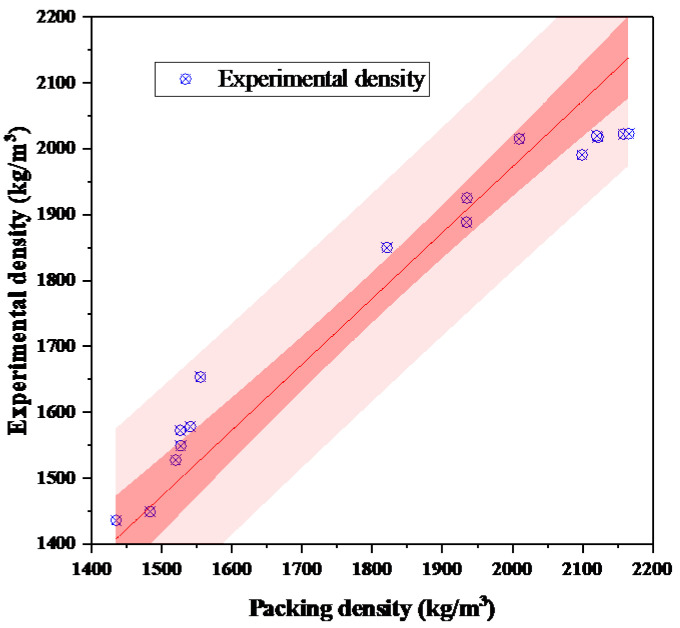
Comparison between predicted and measured packing densities.

The packing densities of the 16 groups of aggregates were calculated using the improved CPM model, and the calculated and experimental values are presented in Figure 2.

The comparison shows that the calculated packing densities from the improved CPM model exhibit a high degree of agreement with the experimental results, with a coefficient of determination R^2^ = 0.90. Most mixtures showed errors below 5%, and 12 out of the 16 tested cases had errors less than 4%. The maximum observed deviation was 6.6%, occurring in mixtures with a high content of fine particles and angular manufactured sand, which are more sensitive to inter-particle interaction assumptions.

To evaluate the practical implications of this error range, it is important to note that even at the upper error bound (6.6%), the predicted paste demand would deviate by less than 1.5% absolute volume in a typical concrete mix design. This level of deviation is within acceptable engineering tolerances, particularly in early-stage proportioning or parametric optimization.

In addition, a sensitivity analysis was conducted by varying two key input parameters: the shape factor *K* (in the range of 7.5–8.5) and the inter-particle interaction coefficient *b* (±10% from baseline). The results showed that the predicted packing density varied within ±2.1% for these parameter changes, indicating the robustness of the model under reasonable input fluctuations. This confirms that the model remains stable and predictive even when aggregate morphology or packing assumptions slightly vary in practice.

These results support the conclusion that the improved CPM model offers reliable predictive performance for multi-fraction aggregate systems and is sufficiently robust for use in practical mix design and optimization.

## 3. Aggregate Surface Area Calculation Model

“Specific surface area” denotes the total surface area of aggregates per unit volume, calculated based on the particle size distribution and morphology. In concrete, after the paste fills the voids between aggregate particles, the excess paste coats the aggregate particles, maintaining a certain distance between them and consequently reducing the probability of inter-particle collisions, thereby improving the workability of the concrete. A larger aggregate surface area leads to a thinner layer of excess paste coating the aggregate surfaces and a smaller inter-particle spacing, which can affect both the workability and mechanical properties of the concrete. Many studies assume aggregate particles to be spherical for specific surface area calculations [20,21,22]:

(6) $$S_{v}=\frac{\pi d^{2}}{\frac{4}{3}\pi r^{3}}=\frac{6}{d}$$

where $S_{v}$ is the specific surface area per unit volume, and d is the diameter of the sphere. For particles within the same size fraction, the particle diameter d is generally taken as the arithmetic mean of the upper and lower limits of the size range. The value of the sphere diameter d is related to the particle size distribution of the aggregates, which varies continuously from the maximum to the minimum particle size.

### 3.1. Effect of Particle Size Distribution

Within each particle size fraction of natural or manufactured aggregates, there exists a range of particle sizes rather than a uniform value. Empirical observations and sieve-based grading curves show that particles within a nominal size class tend to be distributed such that coarser particles are more sparsely spaced than finer ones. This phenomenon aligns with a log-normal or geometric-type distribution, where the relative size difference increases toward the upper bound of the fraction.

To mathematically represent this intra-fraction heterogeneity, we approximate the particle size distribution within each nominal size fraction using a geometric progression, defined as

(7) $$d_{0},d_{0}r,d_{0}r^{2},d_{0}r^{3},\ldots d_{0}r^{n-1},d_{0}r^{n},$$

Here, $d_{0}$ is the minimum particle diameter in the fraction, r is the common ratio, and *n* is the number of discrete sub-particles within the class. This choice is supported by standard sieve size progressions, which follow a geometric ratio, e.g., $r^{n}=2,$ in most international grading systems such as ASTM C136 or ISO 3310-1.

Although other statistical models like Rosin–Rammler or Weibull distributions are also used in powder technology, the geometric model offers analytical simplicity and closed-form expressions for particle volume, surface area, and specific surface area, which are critical inputs in our packing density and paste demand models. Furthermore, this approach is consistent with previous studies on surface-to-volume-ratio prediction in granular systems [23].

The total volume and surface area of the spherical particles in the fraction can be expressed as

(8) $$V=\sum_{i=1}^{n}\frac{\pi d_{i}^{3}}{6},\;S=\sum_{i=1}^{n}\pi d_{i}^{2}$$

Thus, the specific surface area becomes

(9) $$\frac{S}{V}=\frac{6\left[{\left(r^{n}\right)}^{2}-1\right]\left[{\left(r^{n}\right)}^{3/n}-1\right]}{d_{0}{\left(r^{n}\right)}^{1/n}\left[{\left(r^{n}\right)}^{3}-1\right]\left[{\left(r^{n}\right)}^{2/n}-1\right]}$$

Substituting the standard sieve progression ratio $r^{n}$ = 2 yields

(10) $$\frac{S}{V}=\frac{18\left(8^{1/n}-1\right)}{7\left(8^{1/n}-2^{1/n}\right)d_{0}}$$

In the limiting case where *n*→∞, the formula converges to

(11) $$\frac{S}{V}=\underset{n\to \infty }{\lim}\frac{18\left(8^{1/n}-1\right)}{7\left(8^{1/n}-2^{1/n}\right)d_{0}}=\underset{n\to \infty }{\lim}\frac{18}{7d_{0}}\left(1+\frac{1}{2^{2/n}+2^{1/n}}\right)=\frac{27}{7d_{0}}$$

This expression provides a practical estimate of the specific surface area of a given size fraction using only the lower sieve size $d_{0}$, thereby enabling rapid computation for use in our mix optimization model.

### 3.2. Effect of Particle Morphology

The specific surface area of aggregate particles is not only governed by their size distribution but also significantly influenced by their morphological characteristics [24]. Natural aggregates exhibit irregular, non-spherical shapes, leading to deviations when their surface area is approximated using idealized spherical models. To account for these shape effects, we introduce a morphology-adjusted specific surface area model grounded in stereological and image-based measurements.

**Figure 3:**
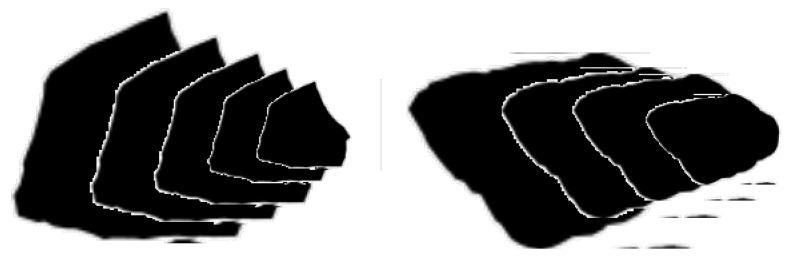
Schematic representation of aggregate projection and bounding geometry.

According to stereological principles, an arbitrary-shaped particle can be geometrically approximated as a stretched double pyramid enclosed within its projected 2D bounding box, as illustrated in Figure 3. This abstraction enables the analytical estimation of surface area and volume based on measurable parameters from two-dimensional images.

In this model, the particle is assumed to rest in its most stable orientation, where its largest projected area ($A_{s}$) serves as the base and the shortest bounding dimension ($D_{min}$) defines the height of the simplified solid. The following equations define particle volume and surface area:

(12) $$V=\frac{A_{s}D_{min}}{6},S=\frac{P_{s}L}{2},\;L=\frac{1}{2}\sqrt{D_{max}^{2}+D_{min}^{2}}$$

Thus, the morphology-adjusted specific surface area of a single particle becomes

(13) $$S_{v}=\frac{3P_{s}}{{2A}_{s}}\sqrt{1+{\left(\frac{D_{max}}{D_{min}}\right)}^{2}}$$

Here, ${2A}_{s}$ = projected area,$\;P_{s}$ = projected perimeter,$\;D_{max}$, $D_{min}$ = long and short axes of bounding rectangle.

To obtain these parameters in practice, digital image analysis was employed. More than 300 particles per size fraction were randomly sampled and imaged using a high-resolution flatbed scanner with uniform lighting. The images were processed using Image Pro Plus (Version 6.0.0.260) software, with a custom macro script to extract key morphological parameters, including equivalent diameter, area ($A_{s}$), perimeter ($P_{s}$), aspect ratio ($D_{max}/D_{min}$D).

Each particle was binarized using adaptive thresholding, and boundary tracing algorithms were applied to ensure accurate perimeter detection. Noise filtering and morphological smoothing were performed to eliminate false contours. The mean values of $A_{s}$, $P_{s}$, $D_{max}$, and $D_{min}$ were calculated for each size group and used to compute an average morphology correction factor.

In addition, to quantify the impact of morphology relative to the ideal spherical model, the shape correction factor was defined as

(14) $$\frac{S_{v}}{S_{v}^{’}}=\frac{{dP}_{s}}{{4A}_{s}}\sqrt{1+{\left(\frac{D_{max}}{D_{min}}\right)}^{2}},where\;S_{v}^{’}=\frac{6}{d}$$

Finally, by combining the morphology-adjusted shape correction factor with the previously derived expression for surface area based on particle size distribution (Equation (11)), the revised specific surface area per unit volume is

(15) $$S_{v}=\frac{{27d\cdot P}_{s}}{{28d_{0}\cdot A}_{s}}\sqrt{1+{\left(\frac{D_{max}}{D_{min}}\right)}^{2}}$$

This formulation enables the practical integration of particle shape effects into mix design optimization models. The large-sample image analysis approach ensures statistical robustness, while the geometric abstraction maintains computational tractability.

### 3.3. Validation of the Specific Surface Area Calculation Model

To validate the accuracy of the proposed morphology-adjusted surface area model, aggregate particles from three representative size fractions—4.75–9.5 mm, 9.5–16 mm, and 16–19 mm—were selected for image analysis. High-resolution images were captured using a flatbed scanner under controlled lighting conditions to ensure consistent resolution and contrast. For each particle size group, at least 300 randomly sampled particles were analyzed to ensure statistical representativeness.

**Figure 4:**
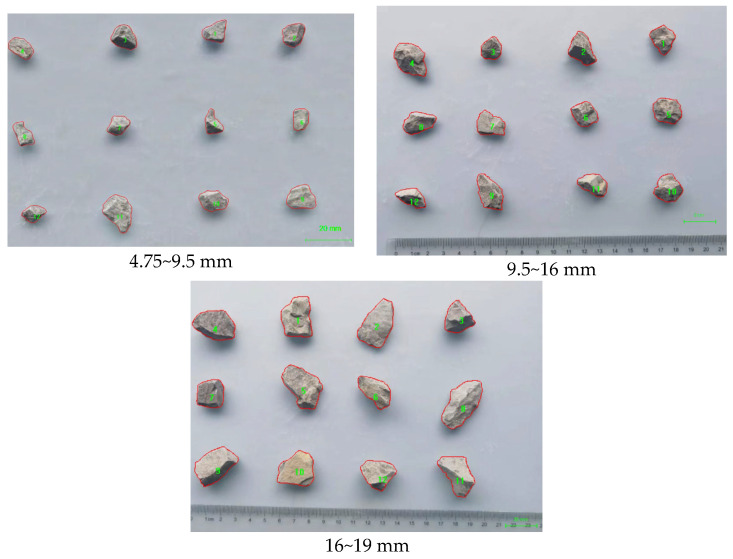
Image analysis of aggregate particles of different particle sizes (The numbers in the image represent the particle count labels).

The projected perimeter $P_{s}$, projected area $A_{s}$, and the major/minor axes $D_{max}$ and $D_{min}$ of each particle were measured using Image Pro Plus (Version 6.0.0.260) software (Figure 4). Noise filtering and contour smoothing were applied to enhance boundary recognition accuracy. Based on Equation (15), the specific surface area for each particle was calculated using the morphology-adjusted formula.

**Figure 5:**
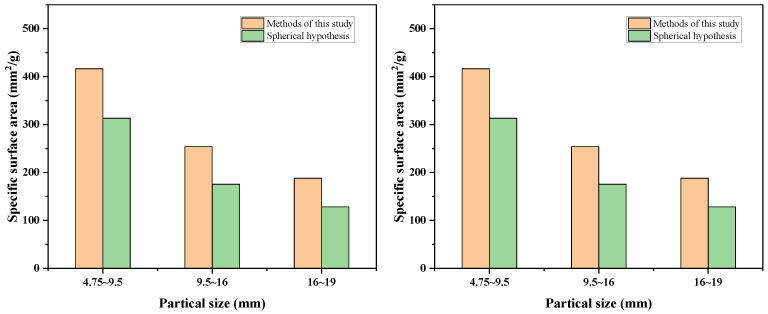
Comparison of specific surface area values calculated by image analysis and spherical model.

For each particle size group, the mean specific surface area $S_{v}$, standard deviation (σ), and 95% confidence intervals were calculated to assess variability and data reliability. A comparison between the measured specific surface area (via image analysis) and the spherical particle assumption is presented in Figure 5.

As shown, the specific surface area values obtained using image-based morphology correction are consistently higher than those calculated under the spherical assumption, indicating that the spherical model underestimates the actual surface area of angular or irregular aggregates. The average enhancement across all fractions was 8.3%, with variability dependent on angularity and aspect ratio of the particles.

For the 9.5–16 mm group, for example, mean $\bar{S_{v}}$ = 4.27 m^2^/m^3^, standard deviation σ=0.18 m^2^/m^3^, 95% confidence interval = [4.25, 4.29] m^2^/m^3^ (based on t-distribution for n = 300).

This analysis confirms that the proposed image-based model provides a more realistic estimate of particle surface area, with quantifiable uncertainty. The narrow confidence intervals across all size groups further support the robustness and repeatability of the methodology.

In summary, image recognition techniques enable the efficient quantification of the aggregate surface area of large samples, thereby addressing a long-standing gap in mix design theory. The quantified morphology-adjusted surface area can be directly used in predictive models to enhance the accuracy of concrete workability and packing behavior assessments, offering an important advancement beyond empirical estimation methods.

## 4. Aggregate Ratio Optimization Method

### 4.1. Concrete Slurry Volume Calculation

In concrete with high workability requirements, a certain amount of excess paste is needed to fill voids and form a lubricating layer between aggregate particles. This concept is quantified in the present model through the definition of the surface-area-based paste thickness (SPT), which reflects the average paste film thickness required to maintain target inter-particle spacing and ensure flowability.

From a packing perspective, the relationship between the void structure and excess paste demand can be expressed as

(16) $$\frac{1-V_{ep}}{\phi }=\frac{1}{\phi_{max}}$$

Here, ϕ is the volume fraction of aggregates in the mixture, and $\phi_{max}$ is the packing density under ideal conditions. Assuming a uniform paste coating on the surface of all particles, the required excess paste volume $V_{ep}$ can also be related to the specific surface area $S_{v}$ and SPT [25]:

(17) $$V_{ep}=1-\frac{\phi }{\phi_{max}}=SPT\times S_{v}\times \phi ,SPT=\frac{\phi_{max}-\phi }{S_{v}\phi_{max}\phi }$$

Substituting into the concrete volume balance gives the expression for total paste demand:

(18) $$V_{p}=1-\phi =1-\frac{\phi_{max}}{S_{v}\phi_{max}SPT+1}$$

In this study, the SPT value was fixed at 25 μm based on the existing literature reporting that a surface paste thickness of 20–40 μm is typically sufficient to ensure pumpability and cohesion for ordinary and high-performance concrete. This fixed value reflects a target workability level and allows the optimization process to focus solely on aggregate proportioning and gradation. The rationality of this SPT value was later evaluated through performance comparisons between optimized and empirical mix designs (see Section 4.3).

It should be noted that the specific surface area $S_{v}\;$and packing density $\phi_{max}$ are not constants but are determined dynamically for each aggregate composition based on the particle size distribution and shape factors (as described in Section 2 and Section 3). By holding SPT constant, the model simplifies to a single-objective optimization problem: minimizing paste volume by adjusting aggregate ratios while maintaining target workability.

### 4.2. Aggregate Gradation Optimization Based on GRC Method

To minimize paste volume while ensuring target workability, an optimization model was constructed based on the paste demand formulation in Section 4.1. For computational clarity, the sand ratio $S_{r}$—defined as the volume ratio of fine aggregate to total aggregate—was used as the single optimization variable. Consequently, both the packing density $\phi_{max}$ and the specific surface area $S_{v}$ can be expressed as functions of $S_{r}$. The objective function becomes

(19) $$\underset{0<S_{r}<1}{\min}V_{p}=f\left(S_{r},\phi_{max}\left(S_{r}\right),S_{v}\left(S_{r}\right),SPT\right)$$

**Figure 6:**
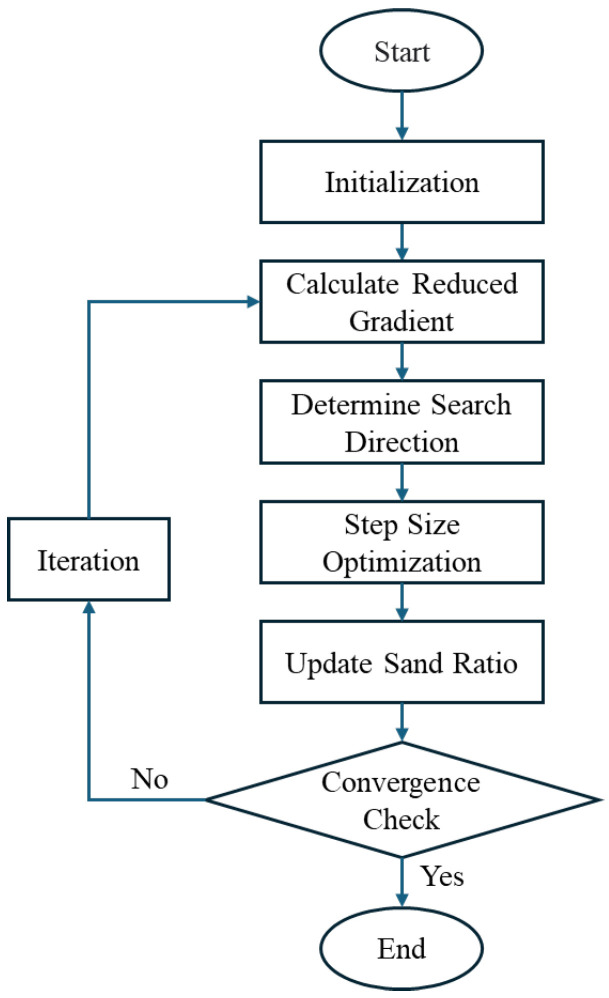
Aggregate gradation optimization process based on GRC method.

We employed the Generalized Reduced Gradient (GRG) algorithm to solve this nonlinear constrained optimization problem (Figure 6) [26]. The GRG method was selected due to its high computational efficiency, numerical stability, and interpretability, being especially suitable for problems with continuous, differentiable objective functions and a low-dimensional design space. Compared with metaheuristic algorithms such as Genetic Algorithms (GAs) or Particle Swarm Optimization (PSO), GRG requires fewer function evaluations and achieves faster convergence when gradients are available and reliable—as is the case in this model.

Figure 6 illustrates the iterative procedure of aggregate gradation calculation using the GRC method. In the preliminary testing, the GRG algorithm consistently converged to stable solutions within a few dozen iterations, whereas the GA required hundreds of function calls to achieve similar paste volumes and introduced slight variability across runs due to stochastic components.

- The optimization begins by assigning a reasonable initial sand ratio $S_{r}^{\left(0\right)}$, typically between 0.30 and 0.45 based on local material knowledge. The algorithm then proceeds iteratively.
- The partial derivative of the objective function $V_{p}$ with respect to $S_{r}$ is computed using central difference approximation.
- The search direction is taken as the negative gradient.
- A line search determines the optimal step size $\alpha^{\left(k\right)}$ that sufficiently reduces the objective value while respecting boundary constraints ${0<S}_{r}<1$.

The updated variable is computed:

(20) $$S_{r}^{\left(k+1\right)}=S_{r}^{\left(k\right)}+\alpha^{\left(k\right)}\cdot d^{\left(k\right)}$$

Convergence is achieved when one of the following conditions is met:

- The relative change in the objective function between iterations is less than 0.001;
- The change in the sand ratio is smaller than 0.0005;
- The maximum iteration count reaches 100.

For all tested cases, the GRG method converged within 15–35 iterations, with an average computation time per optimization run of under 0.5 s on a standard desktop computer (Intel i7 CPU, 16 GB RAM). To assess sensitivity, multiple initial values of $S_{r}$ in the range of 0.30–0.55 were tested. The results showed that the algorithm consistently converged to the same global minimum (±0.2% in paste volume), indicating low sensitivity to initialization and strong robustness.

### 4.3. Experimental Validation and Practical Implications

To assess the effectiveness of the proposed aggregate proportion optimization methodology, eight concrete mixtures were prepared and tested. Four of these were designed using conventional empirical sand ratios, and the other four were optimized using the GRG-based methodology proposed in this study. All mixtures were proportioned to achieve a target slump of 200 ± 10 mm and were evaluated in terms of fresh workability, 7-day and 28-day compressive strength, and paste volume content.

The comparative results are presented in Table 4. On average, the optimized mixes achieved a reduction in paste volume of approximately 10.8% compared to the empirical designs. This reduction was achieved without compromising workability—slump values across all mixes ranged from 195 to 208 mm, meeting the target range. Furthermore, the optimized mixes delivered slightly improved strength performance: 7-day compressive strength increased by an average of 3.1%, and 28-day strength improved by approximately 5.1%

These results clearly demonstrate that the proposed optimization method effectively reduces cement paste demand while maintaining, or even improving, mechanical performance and workability. The use of the surface-area-based paste thickness (SPT) as a control variable proved to be robust, allowing the accurate prediction of the minimum paste requirements to ensure flowability.

Compared to traditional empirical methods, the optimization approach is not only more material-efficient but also more transparent and scalable. It requires only basic input parameters—such as aggregate gradation and shape-based surface area—and provides actionable guidance for practitioners seeking to reduce cement usage without sacrificing performance. The model’s successful application in these eight mixtures confirms its value as a practical tool for low-carbon, performance-driven concrete mix design.

## 5. Conclusions

This study presents a physically grounded and computationally efficient methodology for optimizing aggregate proportions in concrete mixtures based on a binary paste–aggregate framework. By distilling the aggregate system into two measurable descriptors—the packing void ratio and specific surface area—the method enables systematic paste volume minimization while ensuring sufficient inter-particle spacing for target workability.

Using a fixed surface paste thickness (SPT) of 50 μm as a workability constraint, the model guides the optimal adjustment of fine and coarse aggregate ratios. The experimental validation on eight concrete mixtures confirms that, compared to traditional empirical mix designs, the optimized mixes achieve

- A paste volume reduction of up to 13.9%;
- Slump values within ±5 mm of the target (≈200 mm);
- Comparable or improved 7-day and 28-day compressive strengths.

This demonstrates that the proposed approach can significantly reduce cement consumption without compromising fresh or hardened concrete performance, contributing to both cost savings and carbon emission reduction in concrete production.

The model’s strength lies in its physical transparency and operational simplicity. Unlike AI-based approaches that require large datasets and have black-box processing, this method can be applied in data-scarce settings and provides interpretable predictions. Moreover, the optimization process consistently converges with low computational cost (under 0.5 s per run) and shows low sensitivity to initial conditions, further supporting its practical applicability.

However, several limitations must be acknowledged. First, the assumption of a fixed inter-particle spacing may not fully reflect the dynamic particle rearrangements during mixing, casting, or vibration, especially in high-fluidity concretes such as SCC. Second, the model currently focuses on paste volume and workability, without directly incorporating durability metrics such as shrinkage, creep, or long-term permeability. Third, the validation was performed on limited aggregate types and regional material sources; further testing across a broader range of materials is needed.

Future work will aim to

- Extend the framework to multi-objective optimization, incorporating durability and cost–performance trade-offs;
- Integrate real-time sensing or AI modules for adaptive control in field applications;
- Explore dynamic SPT models based on flow simulation or particle tracking to further refine flowability predictions.

In summary, this research bridges the gap between traditional empirical mix design and modern optimization by offering a transparent, quantifiable, and field-ready tool for low-carbon, high-performance concrete production.

## Figures and Tables

**Table 1:** Interaction coefficients between fractions.

	26.5	19	16	9.5	4.75	2.36	1.18	0.6	0.3	0.15	0.075	i
**26.5**	1.000	0.849	0.751	0.486	0.256	0.131	0.066	0.034	0.017	0.008	0.004	bij
**19**	0.851	1.000	0.937	0.646	0.350	0.180	0.092	0.047	0.024	0.012	0.006	bij
**16**	0.782	0.921	1.000	0.741	0.410	0.213	0.109	0.056	0.028	0.014	0.007	bij
**9.5**	0.603	0.712	0.775	1.000	0.646	0.348	0.180	0.093	0.047	0.024	0.012	bij
**4.75**	0.427	0.504	0.549	0.712	1.000	0.643	0.348	0.183	0.093	0.047	0.024	bij
**2.36**	0.301	0.356	0.388	0.503	0.710	1.000	0.646	0.356	0.184	0.094	0.047	bij
**1.18**	0.213	0.252	0.274	0.356	0.503	0.712	1.000	0.655	0.356	0.184	0.094	bij
**0.6**	0.152	0.179	0.196	0.254	0.359	0.509	0.718	1.000	0.646	0.350	0.182	bij
**0.3**	0.107	0.127	0.138	0.179	0.254	0.360	0.509	0.712	1.000	0.646	0.350	bij
**0.15**	0.076	0.090	0.098	0.127	0.179	0.255	0.360	0.504	0.712	1.000	0.646	bij
**0.075**	0.054	0.063	0.069	0.090	0.127	0.180	0.255	0.357	0.504	0.712	1.000	bij
**j**	a_ij_	a_ij_	a_ij_	a_ij_	a_ij_	a_ij_	a_ij_	a_ij_	a_ij_	a_ij_	a_ij_	

**Table 2:** Bulk density of aggregates of each particle size.

Mesh Size/mm	Packing Density/kg·m^−3^	Stacking Compactness
26.5	1354	55.9%
19	1397	57.7%
16	1339	55.3%
9.5	1355	56.0%
4.75	1373	56.7%
2.36	1535	62.0%
1.18	1449	58.5%
0.6	1450	58.6%
0.3	1404	56.7%
0.15	1404	56.7%
0.075	1398	56.5%

**Table 3:** Aggregate bulk density test.

Col.	Coarse Aggregate	Fine Aggregate	Sand	Packing Density Test Value/kg·m^−3^	Calculated Packing Density	Error
1	100.0%	0.0%	0.0%	1483	1449	2.3%
2	0.0%	100.0%	0.0%	1435	1436	0.1%
3	50.0%	50.0%	0.0%	1526	1573	3.0%
4	75.0%	25.0%	0.0%	1527	1549	1.4%
5	62.5%	37.5%	0.0%	1541	1578	2.4%
6	31.3%	68.8%	0.0%	1520	1527	0.5%
7	0.0%	47.1%	52.9%	1935	1925	0.5%
8	0.0%	53.9%	46.2%	1934	1889	2.4%
9	0.0%	77.0%	23.0%	1555	1653	6.3%
10	45.0%	11.0%	44.0%	2010	2015	0.3%
11	44.0%	11.2%	44.8%	2122	2018	4.9%
12	43.0%	11.4%	45.6%	2120	2020	4.7%
13	41.0%	11.8%	47.2%	2158	2022	6.3%
14	39.0%	12.2%	48.8%	2166	2023	6.6%
15	43.0%	17.1%	39.9%	2099	1991	5.2%
16	0.0%	0.0%	100.0%	1821	1850	1.6%

**Table 4:** Comparison of empirical and optimized mix designs.

Mix No.	Design Method	Slump (mm)	7d Strength (MPa)	28d Strength (MPa)	Paste Volume (% vol.)
1	Empirical	198	37.8	45.2	32.2
2	Empirical	202	38.1	46.0	33.8
3	Empirical	200	36.7	43.9	32.9
4	Empirical	195	35.9	42.7	31.5
5	Optimized	203	39.5	47.5	29.4
6	Optimized	208	39.0	47.2	29.1
7	Optimized	198	38.6	46.8	30.5
8	Optimized	200	38.3	46.2	29.3

## Data Availability

The original contributions presented in this study are included in this article. Further inquiries can be directed to the corresponding authors.
